# Genetic diversity, linkage disequilibrium, and population structure of tetraploid wheat landraces originating from Europe and Asia

**DOI:** 10.1186/s12864-023-09768-6

**Published:** 2023-11-14

**Authors:** Ehsan Rabieyan, Reza Darvishzadeh, Reza Mohammadi, Alvina Gul, Awais Rasheed, Fatemeh Keykha Akhar, Hossein Abdi, Hadi Alipour

**Affiliations:** 1 Department of Agronomy and Plant Breeding, University of Tehran, Karaj, Iran; 2 Department of Plant Production and Genetics, Faculty of Agriculture, Urmia University, Urmia, Iran; 3 Dryland Agricultural Research Institute (DARI), AREEO, Sararood branch, Iran; 4 Atta-ur-Rahman School of Applied Biosciences, National University of Sciences and Technology, Islamabad, Pakistan; 5 Institute of Crop Sciences, Chinese Academy of Agricultural Sciences (CAAS), 12 Zhongguancun South Street, Beijing, 100081 China; 6 International Maize and Wheat Improvement Center (CIMMYT), c/o CAAS, Beijing, 100081 China; 7 Department of Plant Sciences, Quaid-I-Azam University, Islamabad, 45320 Pakistan; 8 Department of Plant Biotechnology, College of Agriculture, Jahrom University, Jahrom, Iran

**Keywords:** Asia and Europe durum wheat, Linkage disequilibrium, Genetic variation, Population structure, Single nucleotide polymorphism

## Abstract

**Background:**

Durum wheat is one of the most important crops, especially in the Mediterranean region. Insight into the genetic diversity of germplasm can improve the breeding program management in various traits. This study was done using single nucleotide polymorphisms (SNP) markers to characterize the genetic distinctiveness and differentiation of tetraploid wheat landraces collected from nine European and Asian countries. A sum of 23,334 polymorphic SNPs was detected in 126 tetraploid wheat landraces in relation to the reference genome.

**Results:**

The number of identified SNPs was 11,613 and 11,721 in A and B genomes, respectively. The highest and lowest diversity was on 6B and 6 A chromosomes, respectively. Structure analysis classified the landraces into two distinct subpopulations (*K* = 2). Evaluating the principal coordinate analysis (PCoA) and weighted pair-group method using arithmetic averages (WPGMA) clustering results demonstrated that landraces (99.2%) are categorized into one of the two chief subpopulations. Therefore, the grouping pattern did not clearly show the presence of a clear pattern of relationships between genetic diversity and their geographical derivation. Part of this result could be due to the historical exchange between different germplasms. Although the result did not separate landraces based on their region of origin, the landraces collected from Iran were classified into the same group and cluster. Analysis of molecular variance (AMOVA) also confirmed the results of population structure. Finally, Durum wheat landraces in some countries, including Turkey, Russia, Ukraine, and Afghanistan, were highly diverse, while others, including Iran and China, were low-diversity.

**Conclusion:**

The recent study concluded that the 126 tetraploid wheat genotypes and their GBS-SNP markers are very appropriate for quantitative trait loci (QTLs) mapping and genome-wide association studies (GWAS). The core collection comprises two distinct subpopulations. Subpopulation II genotypes are the most diverse genotypes, and if they possess desired traits, they may be used in future breeding programs. The degree of diversity in the landraces of countries can provide the ground for the improvement of new cultivars with international cooperation. linkage disequilibrium (LD) hotspot distribution across the genome was investigated, which provides useful information about the genomic regions that contain intriguing genes.

**Supplementary Information:**

The online version contains supplementary material available at 10.1186/s12864-023-09768-6.

## Background

Tetraploid wheat (2n = 4x = 28; AABB or AAGG) showed a significant variety in genetic and morphological traits however their evolution under domestication has not been extensively studied or reported yet [1]. The group of tetraploid wheat is relatively divergent and includes species such as *Triticum timopheevii*, *T. araraticum*, *T. dicoccoides*, *T. militinae*, *T. dicoccum*, *T. carthlicum*, *T. polonicum*, *T. ispahanicum*, *T. turgidum*, *T. karamyschevii*, *T. turanicum*, *T. aethiopicum*, and *T. durum* [2]. Durum wheat is the offspring of *Aegilops speltoides* and *Triticum urartu* and became tamed from *Triticum turgidum* ssp. dicoccum in the Fertile Crescent approximately 6000 BC [1, 3–5]. North Africa and the Abyssinian area have been mentioned as the durum wheat secondary center of diversity [6]. Durum wheat plays an important role in food production and is, therefore, one of the most important crops for humans. Durum wheat landraces have a higher genetic diversity than breeding populations [7] and are assumed precious parental germplasm and are used in many wheat breeding programs. Wild relatives and landraces of *Triticum turgidum* are a rich gene pool for agricultural purposes and new sources for the production of modern cultivars [8, 9]. Therefore, investigation of their genetic variation has proved its worth for enhancing and improving Marker-assisted selection in breeding programs [2].

Molecular markers have had a comprehensive application in the study of the genetic and structural heterogeneity of collected or natural germplasms [10–13]. They have critical influences in evaluating variation-related indexes which will lead to facilitating the screening process in breeding programs [14]. As Single nucleotide polymorphisms (SNPs) cover the whole genome of plants, their based markers seem to be the most utilized ones in plant breeding [15]. They are appropriate for the examination of population genetic variation, marker assistant selection (MAS), QTL-based mapping, and map-based cloning which are generally used in plant breeding programs [16].

So far, various molecular markers have been used to study genetic diversity in durum wheat [17–19]. However, the development of high throughput sequencing methods and high-resolution SNP-based maps of wheat in recent years developed its genetic research studies vastly [20–22]. For instance, studying 370 durum wheat samples using an Axiom 35 K array not only separated improved varieties and cultivars but also demonstrated that the Middle East and Ethiopia had the most allelic uniformity among the investigated population [23]. There is another similar report that high genetic diversity in durum wheat landraces [24]. The results of population structure and genetic diversity of a set of durum wheat in the world indicate that breeding programs have different effects on the genomes of this plant [25]. Although it has been concluded that there is an association between the germplasm of durum wheat in some countries [26] and the level of genetic diversity of durum wheat germplasm in some countries is higher than in others [27], further research is needed. Several reports have signified that genotyping based on sequencing has been progressively accepted as a low-cost and high-throughput molecular method for covering full-genome SNPs [20, 28, 29], genotyping, SNP revelation, domestication signature, and genetic variation studies for different plant species covering tetraploid wheat landraces and cultivars [30–33]. Despite the research, the evaluation of the population and genome-wide structure of tetraploid wheat landraces still needs to be assessed using high-throughput SNP genotyping. Covering this gap and studying the genetic structure of tetraploid wheat landraces utilizing a high-density SNP array will be a forward step that will help breeding researchers in conservation and hybridization programs. So this study aimed to investigate the genetic variation and segregation of tetraploid wheat landraces from nine countries using the 55 K Affymetrix SNP Array.

## Results

### The genome SNP distribution of investigated tetraploid wheat

A total of 23,334 polymorphic SNPs were detected in 126 tetraploid wheat landraces with the reference genome. The number of identified SNPs was 11,613 and 11,721 in the A and B genomes, respectively. The amount of identified SNPs varied from 1339 (in chromosome 4 A) up to 2005 in chromosome 5B. The lowest SNP density was observed through chromosome 3B with 1.70 SNP/Mbp and the highest value was found through chromosome 6 A with 3.21 SNP/Mbp, however, the average observed SNP density was 2.34 SNP/Mbp (Table 1).

**Table 1:** A summary of single nucleotide substitutions identified in durum wheat chromosomes and genomes

Chromosomes	1 A	1B	2 A	2B	3 A	3B	4 A	4B	5 A	5B	6 A	6B	7 A	7B	A genome	B genome	Total
No. of SNPs	1762	1582	1463	1977	1492	1425	1339	1426	1679	2005	1978	1573	1900	1733	11,613	11,721	23,334
Chromosome size (Mbp)	585.27	681.11	775.45	790.34	746.67	836.51	736.87	676.29	669.16	701.37	615.67	698.61	728.03	722.97	4857.12	5107.20	9964.32
Density (SNP/Mbp)	3.01	2.32	1.89	2.50	2.00	1.70	1.82	2.11	2.51	2.86	3.21	2.25	2.61	2.40	2.39	2.29	2.34
A↔G	649	585	546	777	584	543	493	538	625	729	748	597	655	645	4300	4414	8714
T↔C	685	626	543	719	527	528	490	529	656	786	754	578	782	643	4437	4409	8846
Transition	1334	1211	1089	1496	1111	1071	983	1067	1281	1515	1502	1175	1437	1288	8737	8823	17,560
Ts %	75.71	76.55	74.44	75.67	74.46	75.16	73.41	74.82	76.30	75.56	75.94	74.70	75.63	74.32	75.23	75.28	75.25
A↔T	34	21	27	26	29	26	27	40	38	35	33	22	28	22	216	192	408
A↔C	152	128	133	142	123	128	127	106	114	153	178	151	157	143	984	951	1935
T↔G	133	114	103	177	136	107	109	120	137	167	130	123	138	156	886	964	1850
C↔G	109	108	111	136	93	93	93	93	109	135	135	102	140	124	790	791	1581
Transversion	428	371	374	481	381	354	356	359	398	490	476	398	463	445	2876	2898	5774
Tv %	24.29	23.45	25.56	24.33	25.54	24.84	26.59	25.18	23.70	24.44	24.06	25.30	24.37	25.68	24.77	24.72	24.75
Ts/Tv ratio	3.12	3.26	2.91	3.11	2.92	3.03	2.76	2.97	3.22	3.09	3.16	2.95	3.10	2.89	3.04	3.04	3.04

Although the number of transition- and transversion-type SNPs was different among chromosomes, the transition/transversion ratio was almost similar in the chromosomes of both genomes. Among observed SNPs, transition types with 75.25% were more than transversion ones (24.75%), while the ratio of transition (Ts) to transversion (Tv) was 3.04 (17,560/5,774) over both genomes (Table 1).

### Genetic diversity and the polymorphism information content (PIC)

**Fig. 1:**
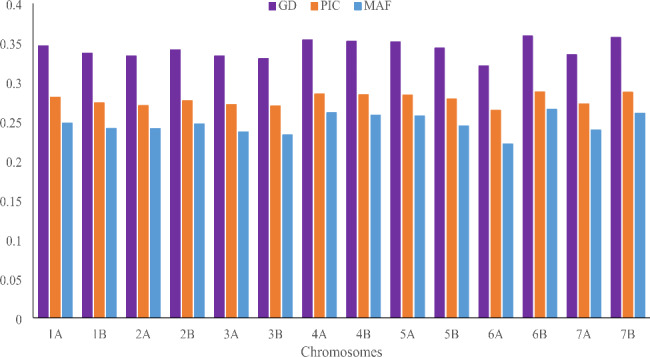
Distribution of gene diversity (GD), polymorphic information content (PIC), and minor allele frequency (MAF) in the different chromosomes for 23,334 SNP markers in the 126 tetraploid wheat landraces

**Fig. 2:**
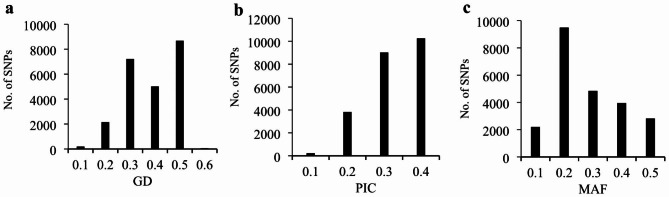
Frequency distribution. Gene diversity (**a**). Polymorphism information content (**b**). Minor allele frequency (**c**)

The maximum PIC values were observed for SNPs on Chromosome 6B (0.29) and minimum on Chromosome 6 A (0.26) (Fig. 1). The gene diversity (GD) and PIC value among all chromosomes ranged from 0.1 (200 SNPs) to 0.6 (65 SNPs) with the average of 0.27 and from 0.1 (228 SNPs) to 0.4 (10,255 SNPs) with an average of 0.46, respectively (Figs. 1 and 2a and b). Approximately 61% of SNPs that covered all chromosomes had PICs greater than 0.25, which relatively implies a high polymorphism for the majority of markers (Fig. 2a and b). More than 90% of SNPs (21,127 SNP) showed a low allele frequency of greater than 0.1 (Fig. 2c). Close values of GD, PIC, and minor allele frequency (MAF) were observed in the chromosomes of wheat. The highest and lowest values of GD, PIC, and MAF were obtained in chromosomes 6B and 6 A, respectively (Fig. 1).

### The relationship and structure of the population

**Fig. 3:**
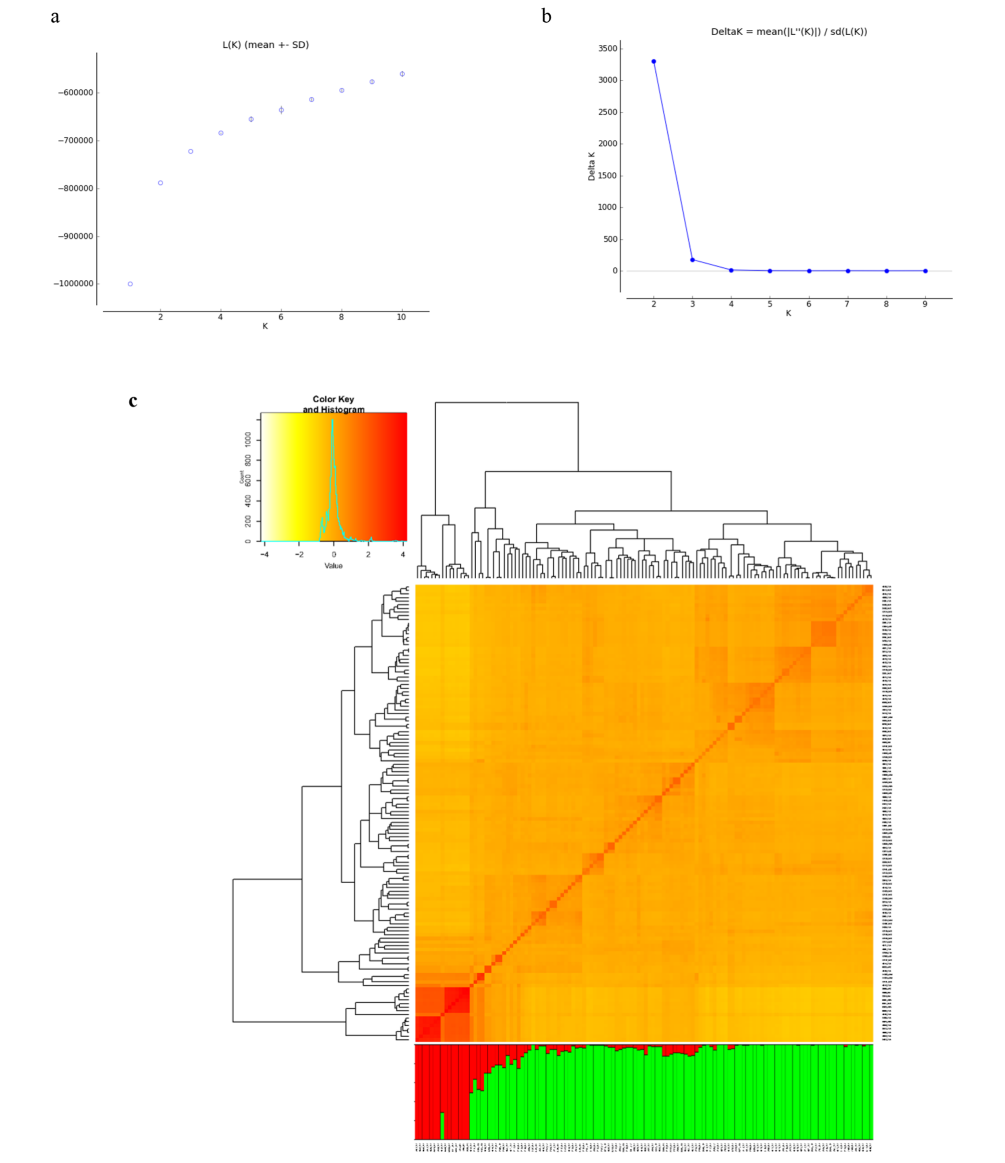
The average log-likelihood value (**a**). Delta K for differing numbers of subpopulations (k) (**b**). Heatmap of pair-wise kinship matrix values and structure plot of the 126 tetraploid wheat landraces determined by K = 2 using 23,334 SNP markers (**c**)

Delta K (ΔK) and log-likelihood [LnP(D)] were utilized to assess the structure of the tetraploid wheat diversity and classify subgroups (K). The evaluated log-likelihood [LnP(D)] showed a gradually increasing value corresponding to the increase of K (Fig. 3a) and the best K value was K = 2, indicating that all 126 investigated tetraploid wheat landraces could be divided into two groups with the highest possibility. Similarly, the largest ΔK was observed at K = 2, confirming two subgroups in the panel (Fig. 3b). The first group consisted of 15 samples, and the second group comprised 111 samples (Fig. 3c). Clustering genetic diversity using kinship matrix also revealed that the association mapping panel was composed of two classes, with significant genetic variation among the landraces (i.e., red to yellow in the heat map clustering output). The pair-wise relative kinship coefficients among the 126 tetraploid wheat landraces ranged from − 0.81 to 4.22. About 68% of the relative kinship values were between zero to 0.05, 26% varied between 0.05 and 0.50, and only 6% were more than 0.50. The heatmap of kinship value showed that most of the values concentrated between zero and 0.05, indicating a weak relatedness in most pairs of tetraploid wheat landraces used in this study (Fig. 3c; ​ Supplementary 1).

**Fig. 4:**
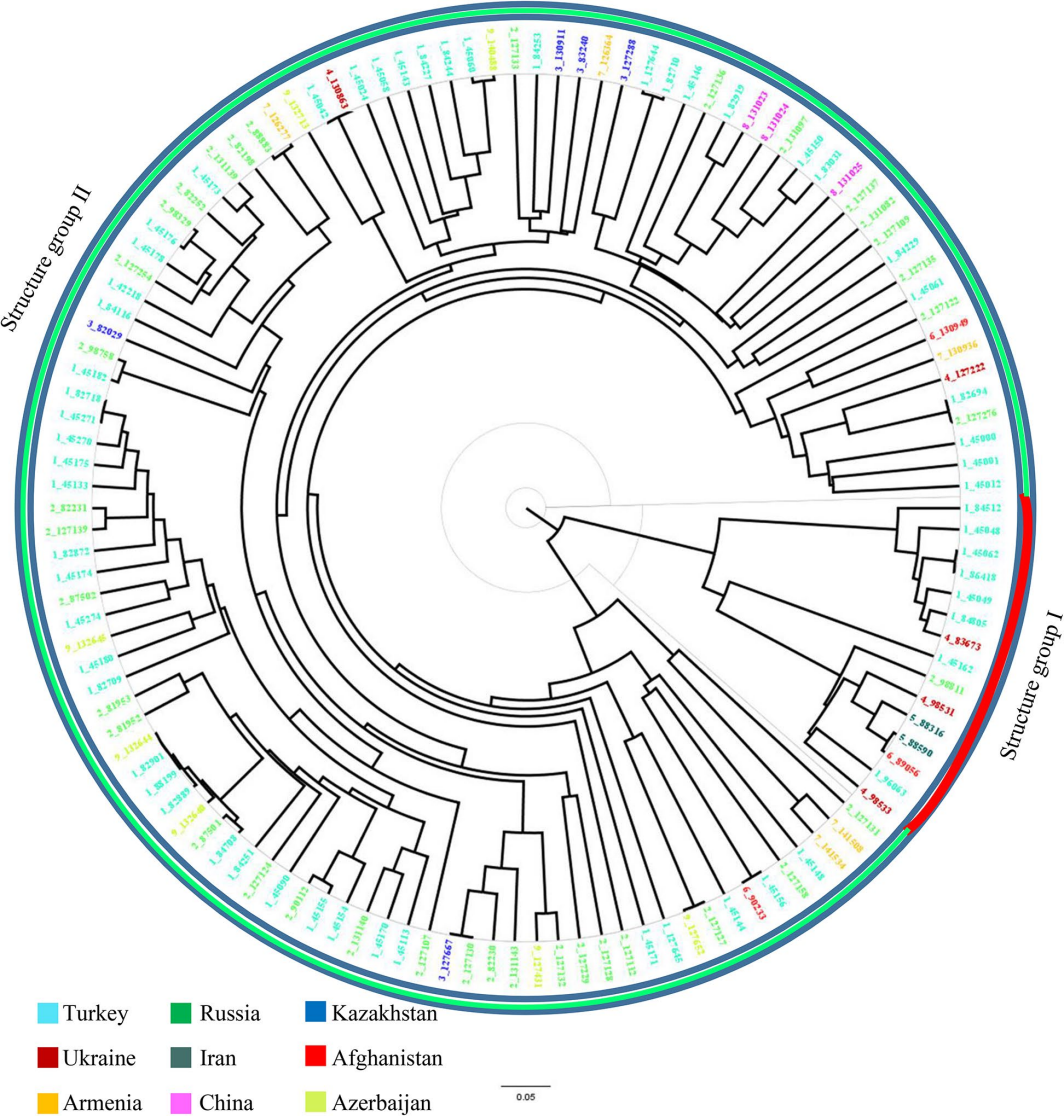
WPGMA clustering dendrogram generated using 23,334 SNP markers and 126 tetraploid wheat landraces. Colors of genotypes code reflect countries of origin

Cluster analysis was also performed using WPGMA to construct a dendrogram from a pairwise similarity matrix (Fig. 4). The WPGMA clustering approach also divided the panel into two classes which were also consistent with observations in structure analysis and the only exception was the genotype 45,148 originated from Turkey. The first main cluster (I) consists of 15 samples including eight samples from Turkey, three samples from Ukraine, two samples from Iran, one sample from Russia, and one sample from Afghanistan. The second main cluster (II) included 111 samples originating in a variety of countries except for Iran.

**Fig. 5:**
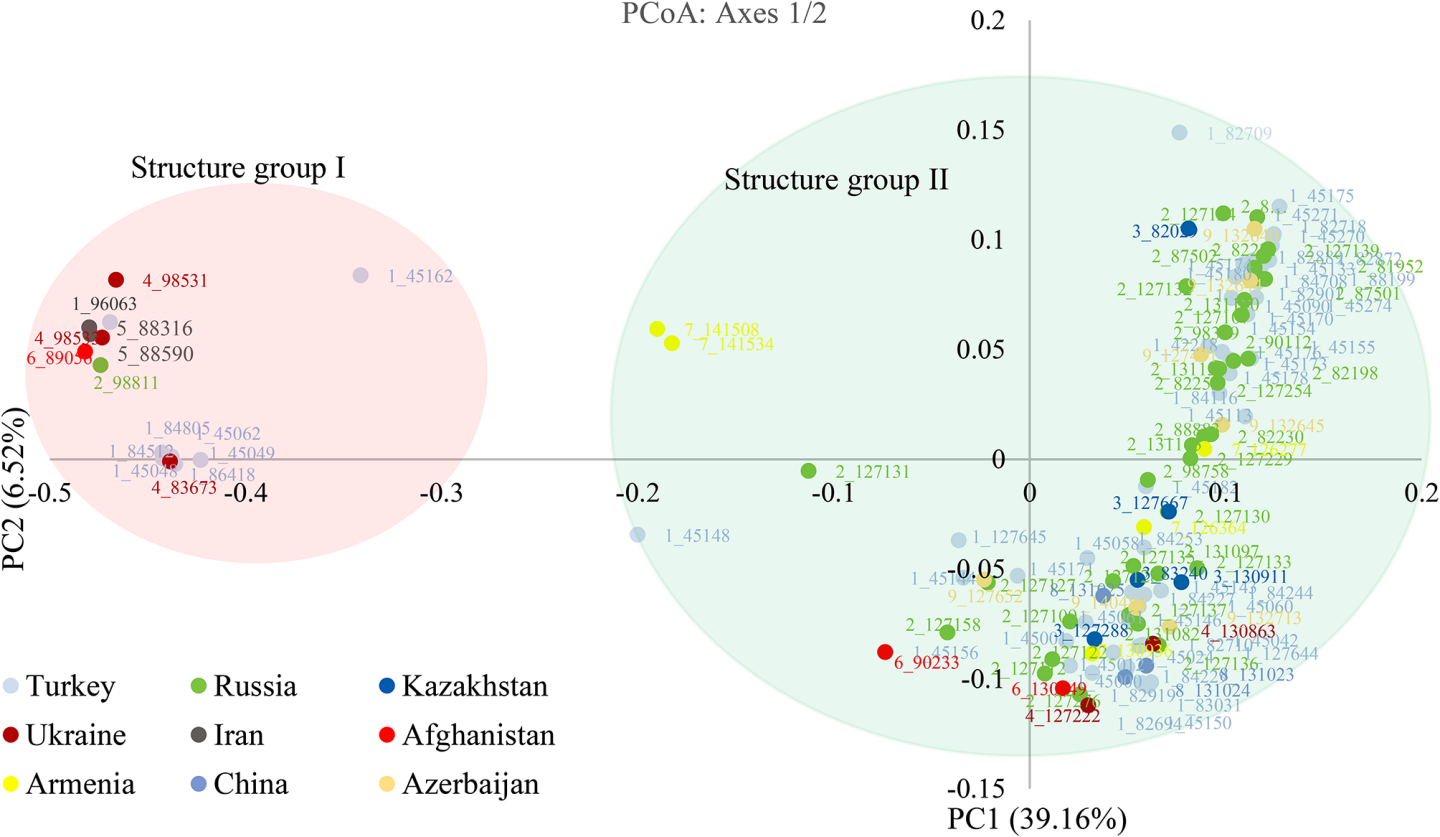
Principal coordinate analysis (PCoA) of 126 tetraploid wheat landraces based on 23,334 SNP markers. Colors of genotypes code reflect countries of origin

**Fig. 6:**
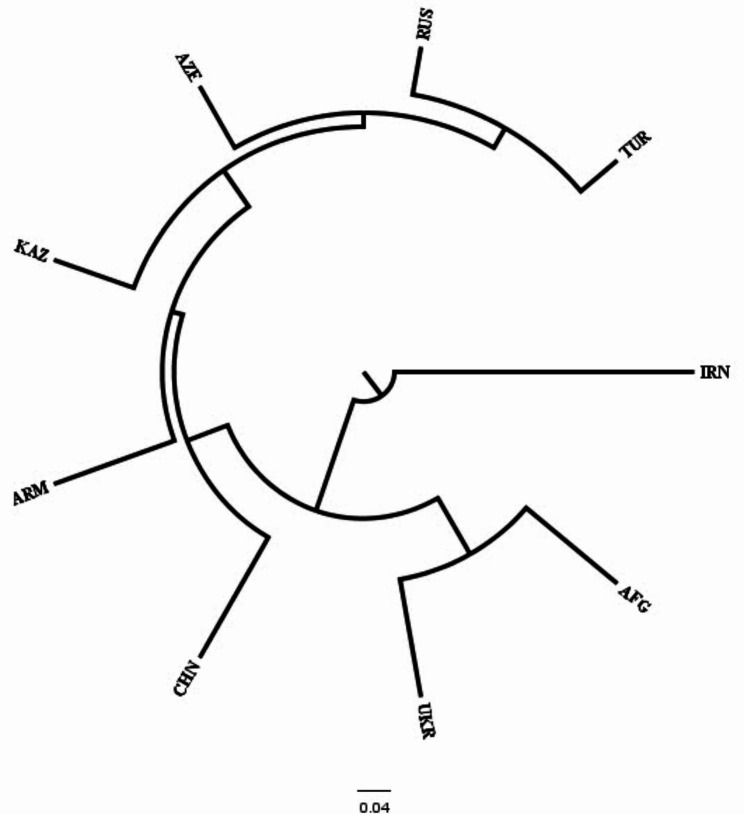
Dendrogram generated using 23,334 SNP markers and 126 tetraploid wheat landraces collected from different countries of origin. *TUR* Turkey, *RUS* Russia, *AZE* Azerbaijan, *KAZ* Kazakhstan, *UKR* Ukraine, *ARM* Armenia, *AFG* Afghanistan, *CHN* China, *IRN* Iran

The results of PCoA were adopted with WPGMA-based clustering results which divided the 126 landraces into two groups (Fig. 5). The first and second coordinates respectively described 39.16% and 6.52% of the total diversity. PCoA1 separated the two groups well so that group I near the origin of the biplot and group II had high negative values (Fig. 5). Genetic variability among the landraces of different countries based on the WPGMA method was shown in Fig. 6. Three clusters were observed: Iran is clearly distinguished from other countries; Afghanistan and Ukraine were delineated in a branch; the remaining countries including China, Armenia, Kazakhstan, Azerbaijan, Russia, and Turkey clustered together.

### Genetic differentiation of populations

The AMOVA was performed based on both different origins and identified two subpopulations in structure analysis (Table 2). The AMOVA result based on the different origin revealed that 9.40% of the whole variations were detected as inter-subpopulations, whereas the remaining variation (90.60%) was classified as intra-subpopulations. However, the AMOVA based on the result of the structure revealed higher variety among the population (53.24%, p < 0.001) than intra-population revealed variation. The fixation index (Fst) of 0.094 among subpopulations from different countries implied a considerable degree of segregation among them whiles a much higher Fst (0.532) between two structure analysis base generated subpopulations implies a great differentiation between the subpopulations. Iran subpopulation showed higher genetic differentiation (Fst) with other subpopulations. After that China subpopulation had higher genetic differentiation (Table 3). Thus, the gene flow between Iran subpopulations with others was much lower than that across the entire range. The highest gene flow was observed between Russia with Turkey (≈ 14.43) and Azerbaijan (≈ 6.27) subpopulations.

**Table 2:** AMOVA analysis of 126 durum wheat landraces

	Source of variation	df	MS	Estimated variance	%	*F statistics (Fst)	Prob.
Region	Among population	8	60132.750	2943.76	9.40	0.094	0.000
	Within populations	117	28358.86	28358.86	90.60		
Structure	Among population	1	794747.91	27581.00	53.24	0.532	0.000
	Within populations	124	24228.23	24228.23	46.76		
	Total of variation	125	30392.384	31302.615			

*df* degrees of freedom, *MS*: Mean of squares

* index considered as a standardized variance of allele frequencies among subdivisions

**Table 3:** Gene flow (Nm, upper diagonal) and pair-wise genetic differentiation (Fst, below diagonal) among durum wheat landraces

	AFG	ARM	AZE	CHN	IRN	KAZ	RUS	TUR	UKR
AFG		1.1776	1.0286	0.7378	0.5383	1.0339	1.4512	1.9010	2.5390
ARM	0.1751		1.5987	0.8401	0.2720	1.4762	2.4467	2.6642	1.2104
AZE	0.1955	0.1352		1.0365	0.2141	2.7125	6.2748	4.9578	0.9652
CHN	0.2531	0.2293	0.1943		0.1295	1.0446	1.9378	1.7378	0.6769
IRN	0.3171	0.4790	0.5387	0.6587		0.2097	0.2867	0.3475	0.8010
KAZ	0.1947	0.1448	0.0844	0.1931	0.5439		4.7721	3.8990	0.9695
RUS	0.1470	0.0927	0.0383	0.1143	0.4658	0.0498		14.427	1.2593
TUR	0.1162	0.0858	0.0480	0.1258	0.4184	0.0603	0.0170		1.8284
UKR	0.0896	0.1712	0.2057	0.2697	0.2379	0.2050	0.1656	0.1203	

*TUR* Turkey, *RUS* Russia, *AZE* Azerbaijan, *KAZ* Kazakhstan, *UKR* Ukraine, *ARM* Armenia, *AFG* Afghanistan, *CHN* China, *IRN* Iran

### The allelic pattern across the populations

Investigated genetic variation within a population based on country grouping demonstrated that average observed (N_a_) and effective (N_e_) allele values were 1.755 and 1.599, respectively (Table 4). The lowest N_a_ (1.093) and N_e_ (1.071) were observed in the Iranian group. The Shannon’s diversity index (I), which varied from 0.06 (Iran group) to 0.53 (Ukraine group). A comparable and close arrangement was seen for expected heterozygosity (Nei’s gene diversity, H_e_) that ranged from 0.041 (Iran group) to 0.368 (Ukraine group). The highest local inbreeding coefficient (F) was found in Ukraine (0.928) and Afghanistan groups (0.925), while the Iran group showed the lowest value of F (-0.362). The percentage of polymorphic loci (PPL) per group varied from 9.66% (Iran group) to 99.96% (Turkey group). Genetic diversity analysis based on the result of structure analysis illustrated that structure group I has a lower value of Na, Ne, I, He, F, and PPL in comparison to structure group II.

**Table 4:** Genetic variation among three groups of 126 durum wheat landraces

Population	N	Na	Ne	I	Ho	He	uHe	F	PPL
Afghanistan	3	1.754	1.598	0.476	0.020	0.332	0.400	0.925	75.41%
Armenia	5	1.690	1.521	0.419	0.029	0.289	0.323	0.875	69.03%
Azerbaijan	7	1.651	1.392	0.349	0.037	0.232	0.251	0.811	65.15%
China	3	1.343	1.273	0.217	0.011	0.151	0.182	0.917	34.33%
Iran	2	1.093	1.071	0.060	0.057	0.041	0.057	-0.362	9.66%
Kazakhstan	5	1.612	1.401	0.345	0.037	0.233	0.260	0.793	61.15%
Russia	37	1.991	1.462	0.438	0.036	0.282	0.286	0.864	99.14%
Turkey	59	2.000	1.554	0.509	0.039	0.335	0.338	0.873	99.96%
Ukraine	5	1.864	1.665	0.530	0.022	0.368	0.409	0.928	86.44%
Average	126	1.755	1.599	0.477	0.020	0.333	0.401	0.926	66.70%
Structure group I	16	1.797	1.434	0.378	0.043	0.250	0.258	0.762	79.69%
Structure group II	110	1.983	1.457	0.430	0.035	0.278	0.279	0.847	98.32%
Average	126	1.890	1.446	0.404	0.039	0.264	0.269	0.809	89.01%

*N* Sample size, *Na* average number of alleles, *Ne* effective number of alleles, *I* information index, *Ho* observed heterozygosity, *He* expected heterozygosity, *F* fixation index, *PPL*: Percentage of polymorphic loci

### Evaluation of linkage disequilibrium

**Fig. 7:**
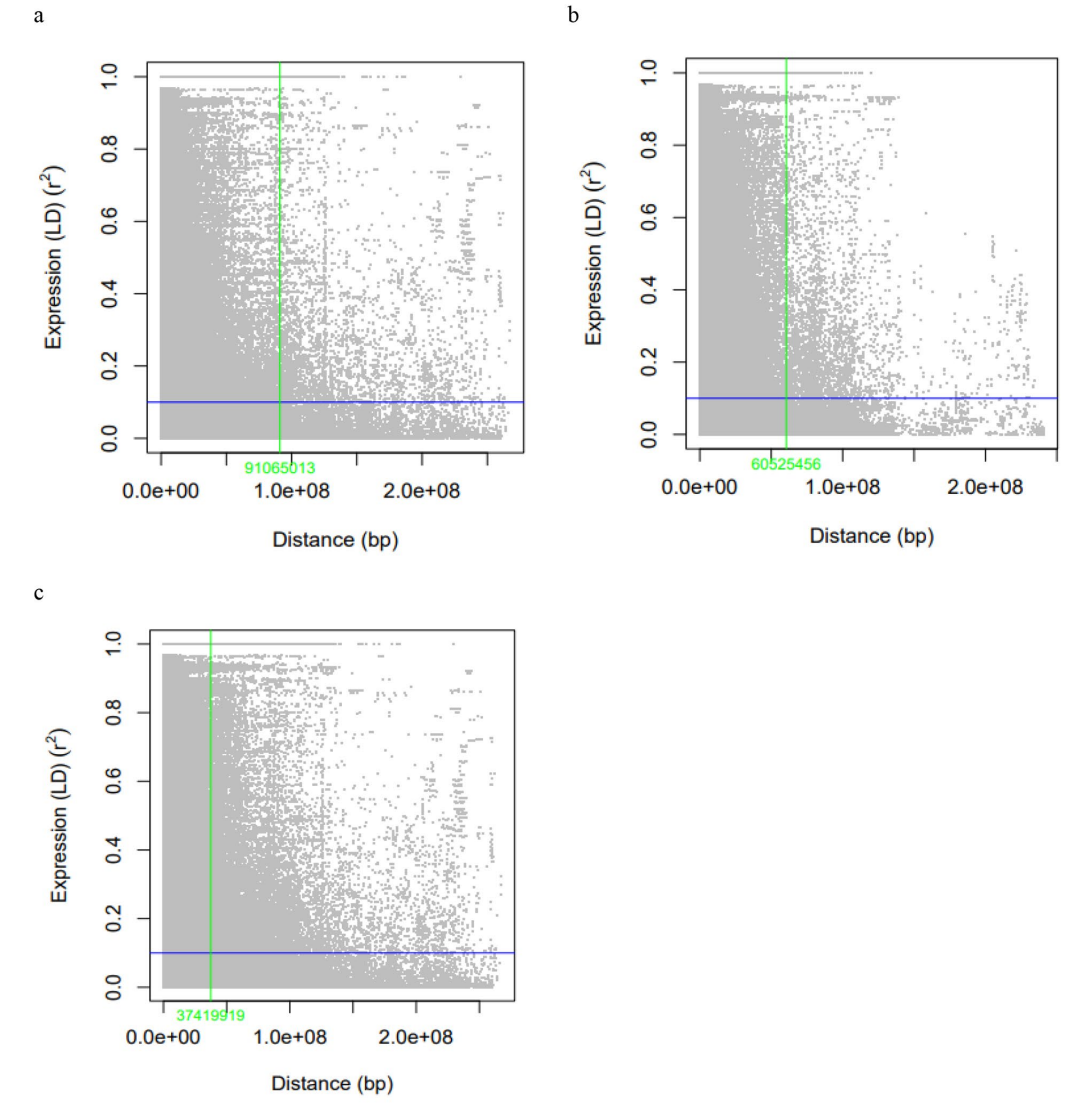
The rate of linkage disequilibrium (LD) decay of the genome A (**a**), genome B (**b**), and total (**c**) of the 126-tetraploid wheat based on the 23,334 SNP markers

Based on the analysis of linkage disequilibrium, it was found that LD decayed with genetic distance. The 23,334 pairs of SNPs in the tested genotypes showed an average R^2^ value of 0.224, suggesting no high LD (Table 5). We found that finding the average of the LD in each genome, rather than measuring the LD between two SNPs located on the same chromosome, was more useful for identifying the pattern of LD across the two genomes. Table 5 represents the average LD/chromosome and the total number of SNP pairs and the number of significant SNP pairs located on the same chromosome. At the genome level, with an average of 0.2501, the A genome had the highest LD, while the B genome had an LD of 0.1978. The LD within each genome ranged from 0.181 (2 A) to 0.423 (6 A) and 0.133 (1B) to 0.242 (5B). The majority of significant marker pairs were located at a distance of < 13,000,000 bp, based on our observations. The A and B genomes possessed the highest number of significant marker pairs (262,768) and least the number (222,240), respectively (Table 5). A diagram showing the LD decay in each genome and over the whole genome is presented in Fig. 7. As compared to the B genome, the A genome showed slower LD decay (Fig. 7). An analysis of the haplotype blocks in the three highest chromosomes was carried out. A total of 11 haplotype blocks were found on chromosome 6 A, while 7 and 8 blocks were found on chromosome 3 A and 5B, respectively (Supplementary 2).

**Table 5:** Linkage disequilibrium between SNP markers located on the same chromosome and genome

Chromosome	TNSP	Distance (bp)	R^2^	NSSP
1 A	86,825	11370559.65	0.22972	37,204
2 A	73,150	14049296.35	0.181004	25,076
3 A	74,600	14856684.32	0.266808	39,038
4 A	66,950	14730561.83	0.185262	24,488
5 A	83,950	11987482.91	0.207666	36,158
6 A	98,900	10173140.3	0.423782	59,644
7 A	95,000	10316144.68	0.214312	41,160
A genome	579,375	12258004.14	0.250612	262,768
1B	77,825	13511157.03	0.133828	21,012
2B	98,850	10470784.78	0.20415	40,642
3B	71,250	15795586.64	0.208633	28,228
4B	71,300	13571325.82	0.241299	33,363
5B	100,250	9603395.024	0.242101	43,296
6B	78,650	13029980.44	0.175531	25,737
7B	86,650	11826634.98	0.17229	29,962
B genome	584,775	12298645.09	0.197803	222,240
Total genomes	1,164,150	12278418.87	0.224085	485,008

TNSP: Total number of SNP pairs, NSSP: Number of significant SNP pairs (p-value < 0.001)

## Discussion

The suitability of SNP markers for the study of genetic diversity and population structure of durum wheat has been proven [24, 25]. Hence in this study, we used a new SNPs array to conduct a genome-wide SNP diversity in tetraploid wheat landraces. The higher proportion of identified SNPs in the B genome is compatible with previously reported results [34, 35]. Although, interestingly chromosome 3B had the lowest SNP density (1.7), Marcotuli et al. [36] also observed the lowest number of mapped markers on chromosome 3B. The abundance of transition-type SNPs is usually detected in true SNPs and reflects the abundance of transition of cytosine to thymine via deamination of 5-methylcytosine after methylation of cytosine [37]. The observed value of Ts/Tv ratio in this study is much higher than what has been reported previously about wheat [20, 29, 32, 38–40] which indicates the higher methylation rate in the genome of durum wheat.

Genetic diversity and PIC values are useful parameters to measure polymorphism among genotypes used in breeding programs. The PIC values for multi-locus markers, such as SSR markers, usually range from 0 to 1.0. Based on their PIC values, Botstein et al. [41] classified multi-allelic markers into three categories. A highly informative marker is one with a PIC value higher than 0.5, a moderately informative marker has a PIC value between 0.25 and 0.5, and a slightly informative marker has a PIC value less than 0.25. The average PIC values of our study were greater than the PIC value reported by Ren et al. [30] and Alemu et al. [24] who investigated genotypes of durum sets with an application of SNP markers. It was reported that this PIC value is a good indicator of informative markers that can be used in studying the genetic diversity of various organisms [42]. Whereas, Mazzucotelli et al. [27] and Baloch et al. [43] observed equal and higher PIC values, respectively, using the same marker. Moragues et al. [44] investigated the genetic variation of 63 durum wheat landraces from the Mediterranean countries using amplified fragment length polymorphism (AFLP) and simple sequence repeats (SSR) markers, and reported 0.24 as PIC value obtained using AFLP and 0.70 from microsatellites. As can be seen, in addition to the marker system, the germplasm studied also has a large effect on the PIC value and it is reported that this value in the landraces is equal [27] to or less [24] than the cultivars and modern lines. The presence of landraces with high geographical distribution in the present study is probably the reason for the high PIC value compared to the same study [24] that only studied the durum landraces of a country. Moreover, to the PIC value, the GD and MAF of each marker among the diversity panel were also evaluated. Chromosomes 6 A and 2 A had the lowest of these indicators, which could be due to the impact of breeding programs and selection pressure [25]. Differences in GD and MAF values of durum wheat chromosomes have already been reported, with 2 and 7 A having the lowest values [24]. Our results suggest that these markers were able to explain the genetic diversity in tetraploid wheat based on their PIC values and good distributions of SNP markers studied. They can be used in other genetic studies to identify alleles associated with target traits, including genome-wide association studies.

Structure analysis classified the landraces into two main subgroups (K = 2). The membership coefficient of 97% (122 out of 126) of samples was higher than 0.7. The multivariate methods including WPGMA clustering, PCoA, and Bayesian model-based clustering approach realized in STRUCTURE software were successful to assign landraces (99.2%) to one of these two primary subpopulations. However, as in the studies of Marzang et al. [45] and Salsman et al. [7], in some clusters, durum wheat landraces were expected to be grouped similar to the geographical pattern. The result of structure analysis, PCoA and WPGMA clustering did not separate landraces based on their region of origin. Therefore, the grouping pattern did not clearly show the presence of a clear pattern of relationships between genetic diversity and their geographical derivation. Part of this result could be due to the historical exchange between different germplasms and has been reported in several studies [19, 24, 30, 46]. As genetic distance plays a very important role in selecting parents for breeding programs, this information is crucial for selecting the candidate parents. It may be unwise to use such parents in breeding programs due to the very low genetic diversity between two genotypes from two different countries, representing two different continents. There is a very important need to understand how the tested 126-tetraploid wheat genotypes relate to each other in terms of population structure. GBS-derived SNPs may be better associated with the studied trait in genome-wide association studies (GWAS) if this is taken into account [47].

In clustering based on countries of origin, it was observed that Iran is completely different from the others and showed lower gene flow and higher genetic differentiation from other countries. Baloch et al. [43] also revealed that the Syrian and Turkish durum wheat landraces are classified into the same group. They indicate that about a hundred years before, there was no obvious breeding program according to the local consumer requirements in those regions. Bousba et al. [48] reported no particular associations between genetic diversity and geographic derivation of durum wheat collections from various countries. Similarly, Haile et al. [49] also evaluated a population consisting of 58 accessions and an advanced improved variety of tetraploid wheat using 31 neutral SSR markers and observed low variability among the released cultivars. Therefore, the dispersal and exchange of seeds among neighboring durum wheat-growing regions could also contribute to the observed higher within-population variation. This result was in accordance with the reported results [49–51]. Moragues et al. [44] indicated the development of the Arabian Empire throughout the Middle Ages as a possible cause of the distribution of germplasm among various regions of the Mediterranean leading to the greater intra-population variation. Availability of multiple wheat ancestral populations may lead to a mixture of landraces’ alleles from multiple gene pools of Mediterranean tetraploid wheat accessions which this process itself has led to combined the admixture of that wheat [52]. Another possible process could be the gene flow among different varieties because of the introduction of new genotypes into fields. It is clear that there is a lower association between genetic differentiation and geographical regions. Some other factors along with geographical origin can affect genetic differentiation among durum wheat landraces. However, Ren et al. [30] illustrated that environmental factors including temperature, and water-accessibility aspects, individually or in composition along with geographical elements, described a critical portion of SNP variation frequency in wild emmer illustrated a vast range of environmental circumstances. The diversity indices values for Iranian durum wheat landraces were very low, which has already been confirmed and it is necessary to expand the genetic basis of durum wheat in Iran [45]. Also, the negative F value in the Iran group indicates more heterozygotes than expected heterozygosity and excess outbreeding. Durum wheat from Turkey and Russia showed the highest diversity. Afghanistan also had good diversity despite the low sample size. Differences in the genetic diversity of durum wheat in different countries are common [25, 27], and this underscores the need for international cooperation to improve new cultivars.

AMOVA revealed that the two subpopulations had highly significant genetic diversity. Due to the selective breeding of specific traits that wheat breeders have done in different countries, subpopulations can show high levels of genetic diversity. Additionally, each subpopulation possessed wheat genotypes from different countries. A low genetic diversity in the populations might be attributed to the spread of wheat germplasm between different regions. As a result, selecting genotypes as parents, for the purpose of improving target traits, from the same subpopulation may be more effective than selecting genotypes from different subpopulations. The incorporation of haplotypes from different founder populations may require crosses between genotypes from different subpopulations. Both winter wheat and synthetic wheat genotypes had high genetic diversity within subpopulations but low genetic diversity among subpopulations [53, 54]. The gene flow level between subpopulations was determined by calculating The haploid number of migrants (Nm). In general, the Nm (haploid) value of 1.00 or lower indicates a low level of gene flow [55]. We observed a very high level of gene flow between the subpopulations in our tested materials with Nm (haploid) of 2.300. This result supports the distribution of the genotypes from one country in the two subpopulations in the tested plant material. Based on all the allelic pattern indices (Na, Ne, I, He, F and PPL) among the three subpopulations, subpopulation II is the most diverse subpopulation as it shows the highest values of all the indices. As a result, this subpopulation is expected to have genotypes from different countries compared to the other subpopulation.

It is essential to determine the magnitude and decay of LDs as they affect the SNP markers and the resolution of association mapping necessary to conduct association studies [56]. There is a wide variation in the extent of LD in different genomes across different species. LD decay in wheat was analyzed separately for each of its two genomes. Based on the nonlinear logarithmic trend line, the LD decay was estimated when LD values declined below 0.1. The LD decayed in genome B at higher distances than in genome A. The lowest rate of LD decay was observed in Ch. 1B. As a result of this finding, the use of GWAS is required for detecting QTLs located in genome B with fewer markers than for QTLs located in genome A [57]. There is a high chance of detecting QTLs with large and small effects in the current materials as a result of the high and low LD found across the two genomes [58]. Ayana et al. [59] and Larmer et al. [60] reported the same pattern of LD decay across the two wheat genome. Each genome contained regions with high LD at high genetic distances. High LD regions adjacent to low LD regions are often referred to as LD hotspots. In comparison to genome B, LD hotspot regions were higher in genome A. This means that understanding the structure of LD and how LD hotspot regions are distributed within wheat genomes is very important. In order to determine the density of markers necessary to associate genotypes with agronomic traits, understanding the LD structure is necessary to determine the genetic regions involved in characterizing these traits [56]. LD hotspots provide useful information about the density of markers in the genome. Higher marker density becomes necessary when the recombination rate is high because the likelihood of the LD being broken by a recombination event increases when the QTL and marker are close together [61]. By looking at the LD plot including the two genomes, hotspot genomic regions were clearly found at a high genetic distance and separated the low LD regions (Supplementary 2).

## Conclusions

Estimation of genetic heterogeneity plays a vital role in plant breeding programs. The current study provides a detailed research-based report of the genetic diversity of tetraploid wheat landraces gathered from various countries. The results indicated that there is a lower association between the geographical origins of tetraploid wheat landraces and their genetic differentiation. Therefore, determined genetic diversity and differentiation of durum wheat materials obtained from diverse regions could provide valuable information for expanding the necessary genetic variation of breeding materials, facilitating and more efficient application of examined wheat resources as selected parental to introduce high-yielding durum wheat genotypes via breeding programs, and associated mapping investigations.

## Materials and methods

### Plant materials

A 126 tetraploid wheat landraces set (Supplementary 3) including accessions from Turkey (n = 59), Russia (n = 37), Azerbaijan (n = 7), Kazakhstan (n = 5), Ukraine (n = 5), Armenia (n = 5), Afghanistan (n = 3), China (n = 3), and Iran (n = 2) were used in current study. These samples were kindly provided by the Dryland Agricultural Research Sub-Institute (DARSI), Agricultural Research, Education and Extension Organization (AREEO), Kermanshah, Iran.

### DNA extraction, genotype-by-sequencing (GBS), and SNP calling

Genomic DNA of samples were extracted using modified CTAB procedure [61] from 2-weeks-old plantlets with 5 replications for each cultivar. DNA concentration was measured by Quant-iTTM PicoGreen® dsDNA Assay (Life Technologies, Inc., Grand Island, NY, United States) and normalized to 20 ng/µl for library construction. The Affymetrix 55 K genotyping Array (CapitalBio Technology Company - Beijing, China) was used for genotyping qualified DNA based on the Axiom® 2.0 Assay for 126 Samples User Manual. low-quality SNPs (score < 15) were eliminated, and SNPs with heterozygosity < 10%, minor allele frequency (MAF) > 10%, and lacking data < 10% were selected as experimental samples for further analysis. Aligning of SNP flanking sequence to the reference genome (Chinese Spring cv.) carried out according to BLASTn analysis using IWGSC ver. 1.0.

### Data analysis

#### Genetic properties of markers

The polymorphic information content (PIC), minor allele frequency (MAF), percentage of heterozygosity, and gene diversity of all 23,334 SNP markers were calculated using PowerMarker software V 3.25 [62]. To calculate the PIC, we used the following formula [41].

$$PIC=1-{\sum }_{j=1}^{n}{P}_{ij}^{2}-{\sum }_{j=1}^{n=1}{\sum }_{k=j+1}^{n}2{P}_{ij}^{2}{P}_{ik}^{2}$$

Where P_ij_ and P_ik_ are the frequencies of j_th_ and k_th_ alleles for marker i, respectively.

### Analysis of population structure

STRUCTURE version 2.3.4 was also used for analyzing the structure of the population based on Bayesian cluster analysis [63] while all parameters were set as their default values, in this situation, the analysis of structure was run 10 times per every K value (K = 1 to 10) applying 30,000 steps for MC and burn-in period and an admixture model [58]. An ad hoc statistic ΔK, based on the change rate of the data log probability of successive K values, was used to estimate the best-fit probability of every hypothetical cluster (K) [64]. Investigated samples with the probability of membership ≥ 0.50 were assigned to corresponding groups [65].

### Analysis of molecular variance (AMOVA) and genetic diversity indices

Genetic variation assessment was carried out with DARwin version 6.010 software [66] based on the Jaccard index. WPGMA and the Neighbor-Joining algorithm [67] were also used to build the diversity. This algorithm produces unrooted trees by assuming mutation rates over time and space equally. To determine the confidence of genetic distance among investigated individuals, 1000 bootstraps were performed which the results are indicated as percent values at the main nodes of each branch. To divide calculated genetic differences into intra- and inter-gene pool groups, Analysis of molecular variance (AMOVA) was done using the pegas package in R software [68].

### Linkage disequilibrium (LD) structure

LD between SNPs in TASSEL V.5 was estimated by using observed/expected allele frequencies. LD distribution was estimated for each subpopulation and for the whole association panel (WAP) using the full matrix option. Due to its less sensitivity to marginal allele frequencies, the pairwise LD was calculated using the squared correlation coefficient of alleles (r^2^). In addition, LD decay was calculated for each chromosome and sub-genome based on the theoretical expectation of r^2^ (see [69] for details).

### Haplotype block analysis

The number of haplotype blocks in each genome was determined using Haploview 4.2 software on the chromosome with the highest significant LD percentage [70]. This was done using SNP data from the target chromosome for calculating pair-wise LD between SNPs. In order to construct these haplotype blocks, the four-gamete method was applied and the cutoff of 1% was used [71–73].

### Electronic supplementary material

Below is the link to the electronic supplementary material.

Supplementary Material 1

Supplementary Material 2

Supplementary Material 3

Supplementary Material 4

## Data Availability

All data generated or analysed during this study are available in Supplementary 4. The sequencing data of 126 accessions used in this study have been deposited into the Figshare repository [10.6084/m9.figshare.23498667].

## References

[1] Matsuoka Y (2011). Evolution of polyploid Triticum wheats under cultivation: the role of domestication, natural hybridization and allopolyploid speciation in their diversification. Plant Cell Physiol.

[2] Sadigov GB, Trifonova AA, Kudryavtsev AM (2017). Genetic diversity in collection of cultivars and varieties of Triticum durum Desf. From Azerbaijan. Russ J Genet.

[3] Salamini F, Özkan H, Brandolini A, Schäfer-Pregl R, Martin W (2002). Genetics and geography of wild cereal domestication in the near east. Nat Rev Genet.

[4] Dvorak J, Akhunov ED (2005). Tempos of gene locus deletions and duplications and their relationship to recombination rate during diploid and polyploid evolution in the Aegilops-Triticum alliance. Genetics.

[5] Feldman M, Levy AA (2005). Allopolyploidy–a shaping force in the evolution of wheat genomes. Cytogenet Genome Res.

[6] Vavilov NI (1951). The origin, variation, immunity and breeding of cultivated plants. LWW.

[7] Salsman E, Liu Y, Hosseinirad SA, Kumar A, Manthey F, Elias E (2021). Assessment of genetic diversity and agronomic traits of durum wheat germplasm under drought environment of the northern Great Plains. Crop Sci.

[8] Taranto F, D’Agostino N, Rodriguez M, Pavan S, Minervini AP, Pecchioni N (2020). Whole genome scan reveals molecular signatures of divergence and selection related to important traits in durum wheat germplasm. Front Genet.

[9] El Haddad N, Kabbaj H, Zaïm M, El Hassouni K, Tidiane Sall A, Azouz M (2021). Crop wild relatives in durum wheat breeding: drift or thrift?. Crop Sci.

[10] Huang X, Börner A, Röder M, Ganal M (2002). Assessing genetic diversity of wheat (Triticum aestivum L.) germplasm using microsatellite markers. Theor Appl Genet.

[11] Dreisigacker S, Zhang P, Warburton ML, Skovmand B, Hoisington D, Melchinger AE (2005). Genetic diversity among and within CIMMYT wheat landrace accessions investigated with SSRs and implications for plant genetic resources management. Crop Sci.

[12] Hao C, Dong Y, Wang L, You G, Zhang H, Ge H, et al. Genetic diversity and construction of core collection in Chinese wheat genetic resources. Sci Bull. 2008;53(10):1518–26. 10.1007/s11434-008-0212-x.

[13] Cavanagh CR, Chao S, Wang S, Huang BE, Stephen S, Kiani S (2013). Genome-wide comparative diversity uncovers multiple targets of selection for improvement in hexaploid wheat landraces and cultivars. Proc Natl Acad Sci.

[14] Astarini IA, Plummer JA, Lancaster RA, Yan G (2004). Fingerprinting of cauliflower cultivars using RAPD markers. Aust J Agric Res.

[15] Batley J, Edwards D. SNP applications in plants. Association Mapp Plants. 2007:95–102.

[16] Kumar S, Banks TW, Cloutier S (2012). SNP discovery through next-generation sequencing and its applications. Int J Plant Genomics.

[17] Robbana C, Kehel Z, Ben Naceur MB, Sansaloni C, Bassi F, Amri A (2019). Genome-wide genetic diversity and population structure of Tunisian durum wheat landraces based on DArTseq technology. Int J Mol Sci.

[18] Marzang N, Abdollahi Mandoulakani B, Shaaf S, Ghadimzadeh M, Bernousi I, Abbasi Holasou H, Sadeghzadeh B (2020). IRAP and REMAP-based genetic diversity among Iranian, Turkish, and International Durum wheat (Triticum turgidum L.) cultivars. J Agric Sci Technol.

[19] Shaygan N, Etminan A, Majidi Hervan I, Azizinezhad R, Mohammadi R (2021). The study of genetic diversity in a minicore collection of durum wheat genotypes using agro-morphological traits and molecular markers. Cereal Res Commun.

[20] Wang S, Wong D, Forrest K, Allen A, Chao S, Huang BE (2014). Characterization of polyploid wheat genomic diversity using a high-density 90 000 single nucleotide polymorphism array. Plant Biotechnol J.

[21] Borrill P, Adamski N, Uauy C. Genomics as the key to unlocking the polyploid potential of wheat. New Phytol. 2015;208(4):1008–22. 10.1111/nph.13533. 10.1111/nph.13533 26108556

[22] Maccaferri M, Ricci A, Salvi S, Milner SG, Noli E, Martelli PL (2015). A high-density, SNP‐based consensus map of tetraploid wheat as a bridge to integrate durum and bread wheat genomics and breeding. Plant Biotechnol J.

[23] Kabbaj H, Sall AT, Al-Abdallat A, Geleta M, Amri A, Filali-Maltouf A (2017). Genetic diversity within a global panel of durum wheat (Triticum durum) landraces and modern germplasm reveals the history of alleles exchange. Front Plant Sci.

[24] Alemu A, Feyissa T, Letta T, Abeyo B (2020). Genetic diversity and population structure analysis based on the high density SNP markers in Ethiopian durum wheat (Triticum turgidum ssp. durum). BMC Genet.

[25] Roncallo PF, Larsen AO, Achilli AL, Pierre CS, Gallo CA, Dreisigacker S (2021). Linkage disequilibrium patterns, population structure and diversity analysis in a worldwide durum wheat collection including Argentinian genotypes. BMC Genom.

[26] Roncallo PF, Beaufort V, Larsen AO, Dreisigacker S, Echenique V (2019). Genetic diversity and linkage disequilibrium using SNP (KASP) and AFLP markers in a worldwide durum wheat (Triticum turgidum L. var durum) collection. PLoS ONE.

[27] Mazzucotelli E, Sciara G, Mastrangelo AM, Desiderio F, Xu SS, Faris J, Hayden MJ, Tricker PJ, Ozkan H, Echenique V, Steffenson BJ (2020). The Global Durum Wheat Panel (GDP): an international platform to identify and exchange beneficial alleles. Front Plant Sci.

[28] Holtz Y, Ardisson M, Ranwez V, Besnard A, Leroy P, Poux G, Roumet P, Viader V, Santoni S, David J (2016). Genotyping by sequencing using specific allelic capture to build a high-density genetic map of durum wheat. PLoS ONE.

[29] Cui F, Zhang N, Fan XL, Zhang W, Zhao CH, Yang LJ (2017). Utilization of a Wheat660K SNP array-derived high-density genetic map for high-resolution mapping of a major QTL for kernel number. Sci Rep.

[30] Ren J, Sun D, Chen L, You FM, Wang J, Peng Y, et al. Genetic diversity revealed by single nucleotide polymorphism markers in a worldwide germplasm collection of durum wheat. Int J Mol Sci. 2013;14(4):7061–88. 10.3390/ijms14047061. 10.3390/ijms14047061 PMC3645677 23538839

[31] He J, Zhao X, Laroche A, Lu ZX, Liu H, Li Z (2014). Genotyping-by-sequencing (GBS), an ultimate marker-assisted selection (MAS) tool to accelerate plant breeding. Front Plant Sci.

[32] Alipour H, Bihamta MR, Mohammadi V, Peyghambari SA, Bai G, Zhang G (2017). Genotyping-by-sequencing (GBS) revealed molecular genetic diversity of Iranian wheat landraces and cultivars. Front Plant Sci.

[33] Maccaferri M, Harris NS, Twardziok SO, Pasam RK, Gundlach H, Spannagl M (2019). Durum wheat genome highlights past domestication signatures and future improvement targets. Nat Genet.

[34] Blanco A, Mangini G, Giancaspro A, Giove S, Colasuonno P, Simeone R, Signorile A, De Vita P, Mastrangelo AM, Cattivelli L, Gadaleta A (2012). Relationships between grain protein content and grain yield components through quantitative trait locus analyses in a recombinant inbred line population derived from two elite durum wheat cultivars. Mol Breed.

[35] Colasuonno P, Gadaleta A, Giancaspro A, Nigro D, Giove S, Incerti O (2014). Development of a high-density SNP-based linkage map and detection of yellow pigment content QTLs in durum wheat. Mol Breed.

[36] Marcotuli I, Gadaleta A, Mangini G, Signorile AM, Zacheo SA, Blanco A (2017). Development of a high-density SNP-based linkage map and detection of QTL for β-glucans, protein content, grain yield per spike and heading time in durum wheat. Int J Mol Sci.

[37] Coulondre C, Miller JH, Farabaugh PJ, Gilbert W (1978). Molecular basis of base substitution hotspots in Escherichia coli. Nature.

[38] Cubizolles N, Rey E, Choulet F, Rimbert H, Laugier C, Balfourier F (2016). Exploiting the repetitive fraction of the wheat genome for high-throughput single‐nucleotide polymorphism discovery and genotyping. Plant Genome.

[39] Winfield MO, Allen AM, Burridge AJ, Barker GL, Benbow HR, Wilkinson PA (2016). High-density SNP genotyping array for hexaploid wheat and its secondary and tertiary gene pool. Plant Biotechnol J.

[40] Rimbert H, Darrier B, Navarro J, Kitt J, Choulet F, Leveugle M, Duarte J (2018). High throughput SNP discovery and genotyping in hexaploid wheat. PLoS ONE.

[41] Botstein D, White RL, Skolnick M, Davis RW (1980). Construction of a genetic linkage map in man using restriction fragment length polymorphisms. Am J Hum Genet.

[42] Salem KFM, Sallam A (2015). Analysis of population structure and genetic diversity of Egyptian and exotic rice (Oryza sativa L.) genotypes. C R Biol.

[43] Baloch FS, Alsaleh A, Shahid MQ, Çiftçi V, Sáenz de Miera E, Aasim L, Nadeem M (2017). A whole genome DArTseq and SNP analysis for genetic diversity assessment in durum wheat from central fertile crescent. PLoS ONE.

[44] Moragues M, Moralejo M, Sorrells ME, Royo C (2007). Dispersal of durum wheat [Triticum turgidum L. ssp. turgidum convar. Durum (desf.) MacKey] landraces across the Mediterranean basin assessed by AFLPs and microsatellites. Genet Resour Crop Evol.

[45] Marzang N, Abdollahi Mandoulakani B, Shaaf S, Ghadimzadeh M, Bernousi I, Abbasi Holasou H (2020). IRAP and REMAP-based genetic diversity among Iranian, Turkish, and International Durum wheat (Triticum turgidum L.) cultivars. J Agric Sci Technol.

[46] Seyedimoradi H, Talebi R, Fayaz F (2016). Geographical diversity pattern in Iranian landrace durum wheat (Triticum turgidum) accessions using start codon targeted polymorphism and conserved DNA-derived polymorphism markers. Environ Exp Biol.

[47] Oraguzie NC, Rikkerink EHA, Gardiner SE, De Silva HN (2007). Association mapping in plants.

[48] Bousba R, Baum M, Djekoune A, Labadidi S, Djighly A, Benbelkacem K, Labhilili M, Gaboun F, Ykhle N (2012). Screening for drought tolerance using molecular markers and phenotypic diversity in durum wheat genotypes. World Appl Sci J.

[49] Haile JK, Hammer K, Badebo A, Nachit MM, Röder MS (2013). Genetic diversity assessment of Ethiopian tetraploid wheat landraces and improved durum wheat varieties using microsatellites and markers linked with stem rust resistance. Genet Resour Crop Evol.

[50] Eujayl I, Sorrells ME, Baum M, Wolters P, Powell W (2002). Isolation of EST-derived microsatellite markers for genotyping the A and B genomes of wheat. Theor Appl Genet.

[51] Abouzied HM, Eldemery SM, Abdellatif KF (2013). SSR-based genetic diversity assessment in tetraploid and hexaploid wheat populations. Br Biotechnol J.

[52] Oliveira HR, Campana MG, Jones H, Hunt HV, Leigh F, Redhouse DI, Lister DL, Jones MK (2012). Tetraploid wheat landraces in the Mediterranean basin: taxonomy, evolution and genetic diversity. PLoS ONE.

[53] Eltaher S, Sallam A, Belamkar V, Emara HA, Nower AA, Salem KFM (2018). Genetic diversity and population structure of F3:6 Nebraska Winter wheat genotypes using genotyping-by-sequencing. Front Genet.

[54] Bhatta M, Morgounov A, Belamkar V, Poland J, Baenziger PS (2018). Unlocking the novel genetic diversity and population structure of synthetic hexaploid wheat. BMC Genomics.

[55] Wright S (1965). The interpretation of population structure by F-statistics with special regard to system of mating. Evolution.

[56] Flint-Garcia SA, Thornsberry JM, Buckler ES (2003). Structure of linkage disequilibrium in plants. Annu Rev Plant Biol.

[57] Liu J, He Z, Rasheed A, Wen W, Yan J, Zhang P (2017). Genome-wide association mapping of black point reaction in common wheat (Triticum aestivum L). BMC Plant Biol.

[58] Würschum T, Maurer HP, Kraft T, Janssen G, Nilsson C, Reif JC (2011). Genome-wide association mapping of agronomic traits in sugar beet. Theor Appl Genet.

[59] Ayana GT, Ali S, Sidhu JS, Gonzalez Hernandez JL, Turnipseed B, Sehgal SK (2018). Genome-wide association study for spot blotch resistance in hard winter wheat. Front Plant Sci.

[60] Larmer SG, Sargolzaei M, Schenkel FS (2014). Extent of linkage disequilibrium, consistency of gametic phase, and imputation accuracy within and across Canadian dairy breeds. J Dairy Sci.

[61] Saghai-Maroof MA, Soliman KM, Jorgensen RA, Allard R (1984). Ribosomal DNA spacer-length polymorphisms in barley: mendelian inheritance, chromosomal location, and population dynamics. Proc Natl Acad Sci.

[62] Liu K, Muse SV (2005). PowerMaker: an integrated analysis environment for genetic maker analysis. Bioinformatics.

[63] Pritchard JK, Wen X, Falush D. Documentation for structure software: Version 2.3. University of Chicago, Chicago, IL. 2010:1–37.

[64] Evanno G, Regnaut S, Goudet J (2005). Detecting the number of clusters of individuals structure: a simulation study using the software. Mol Ecol.

[65] Seyoum M, Du XM, He SP, Jia YH, Pan Z, Sun JL (2018). Analysis of genetic diversity and population structure in upland cotton (Gossypium hirsutum L.) germplasm using simple sequence repeats. J Genet.

[66] Perrier X, Jacquemoud-Collet JP, DARwin. Dissimilarity Analysis and Representation for Windows, Version 5.0. 157. Computer Program. 2006.

[67] Saitou N, Nei M (1987). The neighbor-joining method: a new method for reconstructing phylogenetic trees. Mol Biol Evol.

[68] Paradis E (2010). Pegas: an R package for population genetics with an integrated–modular approach. Bioinformatics.

[69] Remington DL, Thornsberry JM, Matsuoka Y, Wilson LM, Whitt SR, Doebley J (2001). Structure of linkage disequilibrium and phenotypic associations in the maize genome. Proc Natl Acad Sci.

[70] Barrett JC, Fry B, Maller J, Daly MJ (2005). Haploview: analysis and visualization of LD and haplotype maps. Bioinformatics.

[71] Wang N, Akey JM, Zhang K, Chakraborty R, Jin L (2002). Distribution of recombination crossovers and the origin of haplotype blocks: the interplay of population history, recombination, and mutation. Am J Hum Genet.

[72] Mourad AMI, Sallam A, Belamkar V, Wegulo S, Bowden R, Jin Y (2018). Genome-wide association study for identification and validation of novel SNP markers for Sr6 stem rust resistance gene in bread wheat. Front Plant Sci.

[73] Mourad AM, Belamkar V, Baenziger PS (2020). Molecular genetic analysis of spring wheat core collection using genetic diversity, population structure, and linkage disequilibrium. BMC Genom.

