# Supplementary material for: Genetic diversity, linkage disequilibrium, and population structure of tetraploid wheat landraces originating from Europe and Asia
Source: BMC Genomics. 2023 Nov 14;24:682. doi: 10.1186/s12864-023-09768-6 (PMC10644499; doi:10.1186/s12864-023-09768-6)
Supplement: Supplementary file 2 — Supplementary Material 2 [file 12864_2023_9768_MOESM2_ESM.docx]

a


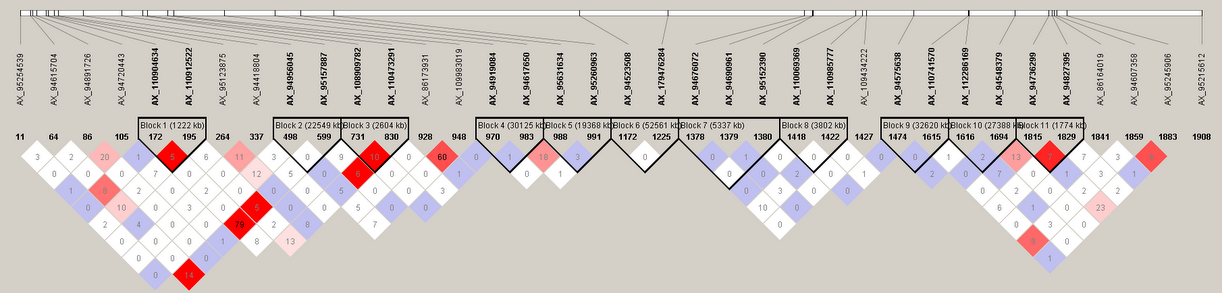


b


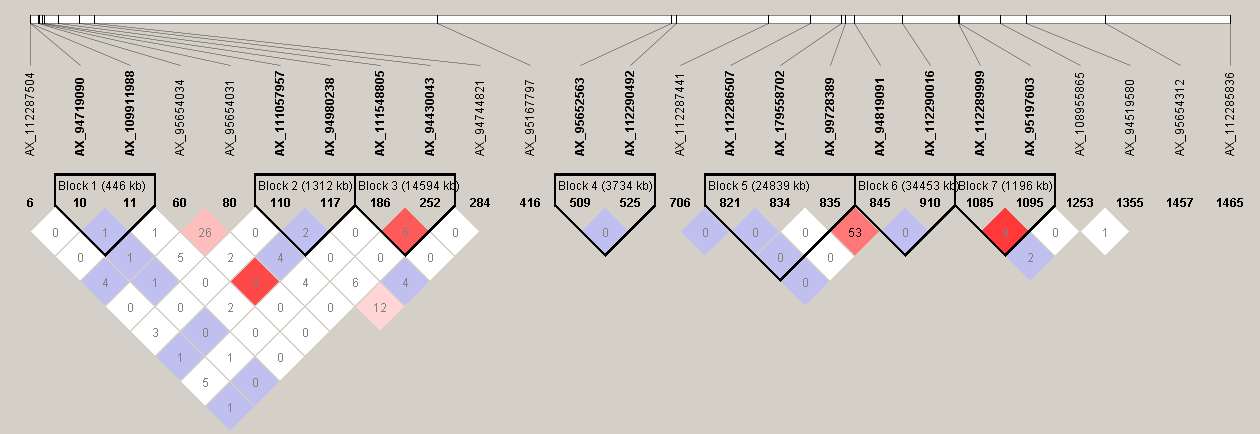


c


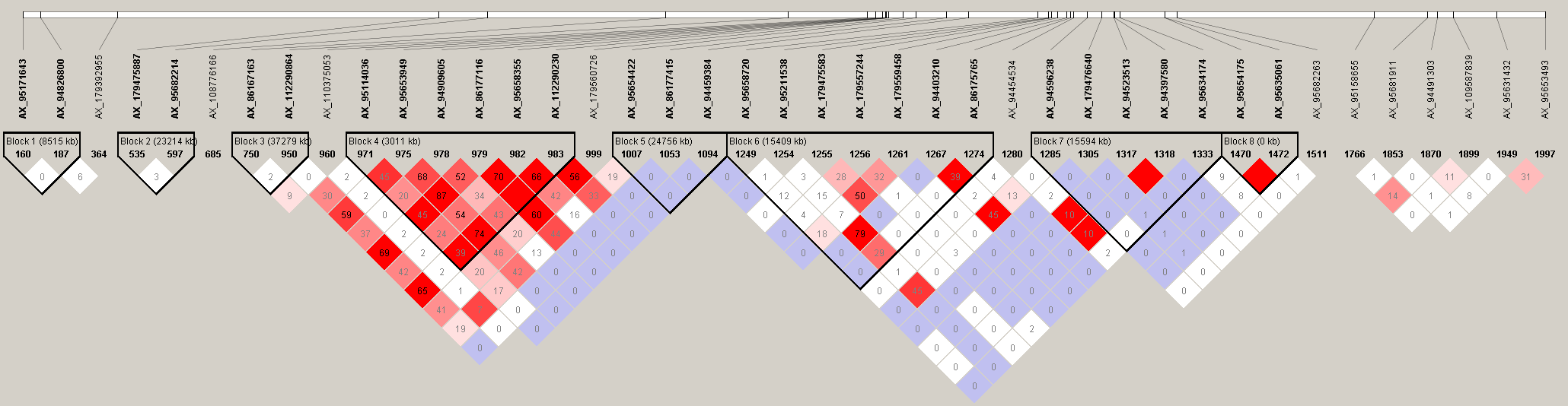


**Supplementary 2**.The number of haplotype blocks in each genome was determined using Haploview 4.2 software ‎on the chromosome with the highest significant LD percentage‎. a chromosome 6A, b chromosome 3A, c chromosome 5B
